# Satisfaction and Experience With a Supervised Home-Based Real-Time Videoconferencing Telerehabilitation Exercise Program in People with Chronic Obstructive Pulmonary Disease (COPD)

**DOI:** 10.5195/ijt.2016.6213

**Published:** 2016-12-15

**Authors:** LING LING Y. TSAI, RENAE J. MCNAMARA, SARAH M. DENNIS, CHLOE MODDEL, JENNIFER A. ALISON, DAVID K. MCKENZIE, ZOE J. MCKEOUGH

**Affiliations:** 1 DISCIPLINE OF PHYSIOTHERAPY, UNIVERSITY OF SYDNEY, LIDCOMBE, NEW SOUTH WALES, AUSTRALIA; 2 DEPARTMENT OF PHYSIOTHERAPY, PRINCE OF WALES HOSPITAL, RANDWICK, NEW SOUTH WALES, AUSTRALIA; 3 DEPARTMENT OF RESPIRATORY AND SLEEP MEDICINE, PRINCE OF WALES HOSPITAL, RANDWICK, NEW SOUTH WALES, AUSTRALIA; 4 NEW SOUTH WALES AGENCY FOR CLINICAL INNOVATION, CHATSWOOD, NEW SOUTH WALES, AUSTRALIA; 5 ALLIED HEALTH, SYDNEY LOCAL HEALTH DISTRICT, NEW SOUTH WALES, AUSTRALIA

**Keywords:** Telerehabilitation, Real-time videoconferencing, Satisfaction, Semi-structured interview, Surveys, Chronic obstructive pulmonary disease, Qualitative

## Abstract

Telerehabilitation, consisting of supervised home-based exercise training via real-time videoconferencing, is an alternative method to deliver pulmonary rehabilitation with potential to improve access. The aims were to determine the level of satisfaction and experience of an eight-week supervised home-based telerehabilitation exercise program using real-time videoconferencing in people with COPD. Quantitative measures were the Client Satisfaction Questionnaire-8 (CSQ-8) and a purpose-designed satisfaction survey. A qualitative component was conducted using semi-structured interviews. Nineteen participants (mean (SD) age 73 (8) years, forced expiratory volume in 1 second (FEV_1_) 60 (23) % predicted) showed a high level of satisfaction in the CSQ-8 score and 100% of participants reported a high level of satisfaction with the quality of exercise sessions delivered using real-time videoconferencing in participant satisfaction survey. Eleven participants undertook semi-structured interviews. Key themes in four areas relating to the telerehabilitation service emerged: positive virtual interaction through technology; health benefits; and satisfaction with the convenience and use of equipment. Participants were highly satisfied with the telerehabilitation exercise program delivered via videoconferencing.

The most effective non-pharmacological management strategy for people with chronic obstructive pulmonary disease (COPD) is pulmonary rehabilitation which decreases breathlessness, improves quality of life and increases exercise capacity (McCarthy et al., 2015). However, major barriers in attending centre-based pulmonary rehabilitation exist including difficulties accessing the venue, cost of travel and lack of mobility (Keating, Lee, & Holland, 2011).

Telerehabilitation is defined as the delivery of rehabilitation services using telecommunication technologies (Lundell, Holmner, Rehn, Nyberg, & Wadell, 2015), such as real-time videoconferencing. A number of pilot cohort studies have demonstrated that telerehabilitation using videoconferencing facilities has a positive effect on exercise capacity, quality of life, self-efficacy and high adherence and completion rates in people with COPD (Holland, 2013; Marquis, 2015; Paneroni, 2015; Tousignant, 2012; Zanaboni, 2013). Some of these studies measured satisfaction levels via self-reported questionnaires (Marquis, 2015; Paneroni, 2015) or a focus group (Hoaas, Andreassen, Lien, Hjalmarsen, & Zanaboni, 2016), with results indicating high levels of satisfaction with telerehabilitation. Other telerehabilitation studies have reported on alternative exercise delivery methods such as online group training and shown these to be feasible for people with COPD (Burkow, 2013; Taylor, 2011). However, no studies have examined the opinions of the partners or carers of people who complete a telerehabilitation program.

The telerehabilitation for people with COPD (TeleR) trial was the first prospective randomised controlled trial to use real-time videoconferencing to supervise home-based exercise training in people with COPD (Tsai, McNamara, Moddel, Alison, McKenzie, & McKeough, 2016). This study showed a positive effect of telerehabilitation on exercise capacity and self-efficacy as well as high adherence rates in people with COPD ( Tsai, McNamara, Moddel, Alison, McKenzie, & McKeough, 2016). This current report is an extension of the TeleR study and aimed to determine the level of satisfaction and experience of an eight week, supervised, home-based telerehabilitation exercise intervention using real-time videoconferencing technology in people with COPD. A secondary aim was to determine the level of satisfaction with telerehabilitation of the partners of people with COPD who completed the telerehabilitation program.

## METHODS

### PARTICIPANTS

Patients who were referred to a tertiary hospital pulmonary rehabilitation program in Sydney, Australia with a primary medical diagnosis of stable COPD (forced expiratory volume in one second (FEV_1_)/forced vital capacity (FVC) <70% and FEV_1_ < 80% predicted post bronchodilator) were invited to participate in the TeleR study (Tsai et al., 2016). Participants were assessed and educated on the use of equipment (laptop computer and stationary cycle) prior to recruitment to ensure that they were competent in using these independently and safely before entering the TeleR study (Tsai et al., 2016). Participants who were randomised to the intervention group (n=20) completed an eight-week supervised home-based telerehabilitation exercise program and were invited to be involved in the quantitative aspects of this study. These participants were then randomly chosen by an independent investigator (random number system) to undergo the qualitative aspects of this study. All partners of the participants who had been involved in the telerehabilitation intervention were also invited to be a part of this study. The study was approved by the South Eastern Sydney Local Health District Human Research Ethics Committee (approval number 12/177) and registered on the Australia New Zealand Clinical Trials Registry (ACTRN12612001263886).

### INTERVENTION

Participants in the telerehabilitation group completed an exercise training program at home consisting of one hour sessions, three times a week for eight weeks, supervised remotely by an experienced physiotherapist who was located at the hospital. Up to four participants were simultaneously supervised in a virtual group using real-time desktop videoconferencing software (VSee, http://vsee.com) via a laptop computer with an in-built camera (HP EliteBook 8560p) connected to a wireless 4G cellular data network. Participants could see and talk to both the physiotherapist and the other participants. All participants performed lower limb cycle ergometry on a stationary cycle ergometer (Tunturi, E60), ground-based walking training within their home, and strengthening exercises for lower limbs. A finger-tip pulse oximeter (Nonin Onyx Vantage 9590) was used by the participants to monitor their oxygen saturation and heart rate throughout the exercise training program. Details of the exercise program, including intensity and progression of training, have been previously published (Tsai et al., 2016). Troubleshooting of equipment use was resolved over the telephone and if not resolvable, the physiotherapist visited the participant’s home. No formal education was provided as part of the program.

### QUANTITATIVE OUTCOMES

One questionnaire and two surveys were used: the Client Satisfaction Questionnaire-8 (CSQ-8) (Appendix A) and two purpose-designed surveys. The CSQ-8, a validated questionnaire assessing client satisfaction with a service (Larsen, Attikisson, Hargreaves, & Nguyen, 1979) and a purpose-designed participant’s satisfaction survey were completed at the end of the eight-week telerehabilitation exercise program when participants returned to the hospital to complete their final assessment with a blinded assessor. The participant satisfaction survey consisted of 20 statements with responses recorded on either a four or five point Likert scale. For ease of interpretation of the analysis the five point Likert scale of “strongly agree” and “agree” as well as “disagree” and “strongly disagree” were collapsed for either positive or negative results. The 20 statements were related to virtual interaction via telerehabilitation equipment (i.e., laptop computer, stationary cycle, pulse oximeter), timeliness and convenience, and level of satisfaction (Table 2). A purpose-designed participants’ partner’s satisfaction survey was completed by participants’ partners in their own home and returned to the investigators either by mail or in person. This survey consisted of seven statements related to the impact of the service on the participant, with responses recorded on a five point Likert scale (Table 3). Both purpose-designed surveys also allowed for open-ended written comments. The surveys were developed with the assistance of the Mode for Assessment of Telemedicine (MAST) Manual (Kidholm et al., 2010).

### QUALITATIVE OUTCOMES

Eleven participants who completed the telerehabilitation exercise program were randomly chosen by an independent investigator to undergo an individual semi-structured interview following the completion of the telerehabilitation exercise program. The interview questions (Appendix 2) extended on the information provided from the participant’s satisfaction survey and gave the opportunity for participants to express their views on the telerehabilitation exercise program in more detail. Interviews were conducted face-to-face by an independent investigator who was not involved in the participants’ assessment and treatment, allowing the participants to express their opinion in a non-biased manner. Interviews were recorded with a digital recorder.

## DATA ANALYSIS

All quantitative data, including participants’ characteristics and the CSQ-8, were analysed using SPSS software (Version 20 for Windows, IBM, USA). Data for the purpose-designed surveys were expressed as percentages for each statement. Semi-structured interviews were transcribed verbatim by a person independent of the research team. Thematic and descriptive qualitative analysis (Braun & Clarke, 2006) was used to identify key themes regarding participants’ satisfaction and perception of their experiences with the telerehabilitation exercise program. Initial coding of key themes using NVivo 9 (QSR International, Melbourne, Vic., Australia) was used to reflect on the interview questions. Two investigators who were not involved in assessing and treating the participants interpreted the data independently to ensure coding was insightful. Any discrepancies were discussed by the research team and the coding revised accordingly.

## RESULTS

Twenty participants were randomised to the telerehabilitation intervention group in the randomised controlled trial. Of these, one participant died due to an adverse reaction to a medication unrelated to the study and the other 19 participants completed the study. All participants except one exercised with at least one other person, commonly in groups of two to four people. Characteristics of these participants, as well as the subset of participants who completed the semi-structured interviews (n=11), are reported in Table 1. Six out of the 11 participants interviewed had previously attended a hospital-based pulmonary rehabilitation program more than two years prior to entering this study. Out of the 19 participants, eight participants lived with their partner and 11 lived alone. Seven participants’ partners (2 male, 5 female) completed the partner survey with mean (SD) age 72 (5). None of the partners were smokers, and three (43%) were employed with the remaining partners being retired. One participant’s partner was too unwell to fill out the survey.

### QUANTITATIVE FINDINGS

#### CLIENT SATISFACTION QUESTIONNAIRE-8 (CSQ-8)

The results of the CSQ-8 showed a high level of satisfaction with the telerehabilitation service amongst participants, with a mean (SD) score of 30 (2) out of a total score of 32.

#### PARTICIPANT SATISFACTION SURVEY

Participants reported high levels of agreement with the positive virtual interaction they had with the real-time videoconferencing, exercising at home using the equipment provided, and the timeliness and convenience of the program (Table 2). The overall satisfaction levels were high (Table 2). The two areas that had the lowest ratings related to the use of equipment and participants’ interaction with others, with two participants stating that they required help while using the computer equipment, and one participant reporting no benefit from interacting with other participants during the sessions.

#### PARTICIPANTS’ PARTNERS’ SATISFACTION SURVEY

Participants’ partners reported a high level of satisfaction overall with the telerehabilitation exercise program (Table 3). The only question that did not have 100% agreement in this survey related to the reliability and ease of use of the exercise equipment and computer, where 28% (n=5) responded “neutral” (Table 3).

#### QUALITATIVE FINDINGS

Key themes in four areas relating to the telerehabilitation service emerged: (a) virtual interaction through technology; (b) use of equipment; (c) convenience; and (d) health benefits.

##### (a) Virtual interactions through technology

All participants reported positive experiences when interacting through real-time videoconferencing technology to perform their supervised exercise training. The lack of direct face-to-face interaction with the physiotherapist was not perceived to be a problem by participants. In fact, positive comments were reported about interacting with the physiotherapist via a computer (Table 4, Quotes 1 and 2). Ten participants (91%) expressed their enjoyment of having others to exercise with through the use of real-time videoconferencing technology, particularly to provide encouragement and support (Table 4, Quotes 3 and 4). One participant was unable to join a group session due to his work hours, however, he did not feel it affected his experience (Table 4, Quote 5).

##### (b) Use of equipment

Ten participants (91%) reported that the equipment was easy to use once the physiotherapist had set it up and demonstrated its use in their home environment, providing opportunity to practice with supervision on one initial occasion (Table 4, Quotes 6 and 7). Eight participants (73%) reported they had adequate space at home for equipment.

The main issue regarding the equipment that was raised in the interviews was difficulty on some occasions with the internet connection (Table 4, Quote 8), with three people (27%) reporting internet connection problems. These participants required the physiotherapist to attend their home on a second occasion to resolve these problems.

##### (c) Convenience

Participants reported the service was very convenient as it saved costs when compared to parking at the hospital and it also saved travel time. In particular, the service worked well for those who were still employed due to the flexibility of the scheduling of the telerehabilitation sessions (Table 4, Quotes 9 and 10).

Participants who had previously attended pulmonary rehabilitation reported that attending the hospital often took more time out of their day and that they often felt quite breathless by the time they arrived at the venue. Other participants had carer responsibilities or co-morbidities that impacted on their lives so exercising from home was more convenient (Table 4, Quotes 11 to 13).

##### (d) Health benefits

Ten participants (91%) said that they felt motivated during the program and experienced positive health benefits after the telerehabilitation exercise program which they related to the supervision of the physiotherapist (Table 4, Quotes 14 and 15). All participants were overall satisfied with the telerehabilitation service (Table 4, Quotes 16 and 17).

## DISCUSSION

This study has reported high levels of satisfaction and positive experiences of an eight-week supervised home-based telerehabilitation exercise program using real-time videoconferencing technology in people with COPD. A strength of this study was the triangulation of quantitative and qualitative outcome measures to understand the participants’ experience of telerehabilitation. Interactions via real-time videoconferencing technology were well accepted by participants; the equipment was easy to use once the physiotherapist had educated participants in their home environment; convenience was a key factor on the positive perception of the program; and participants were very satisfied with the positive health benefits they gained from the program. The only negative aspect reported by participants related to some difficulties with the internet connection but this did not impact on the positive health improvements.

The quantitative results of this study, indicating high levels of satisfaction, are consistent with other pilot studies of telerehabilitation in people with COPD (Hoaas et al., 2016; Marquis et al., 2015; Paneroni et al., 2015). Telerehabilitation is an evolving mode of therapy delivery, therefore different types of questionnaires have been used across the studies. The main focus of the questionnaires were similar, including questions on the patient and healthcare professionals relationship, ease of use of the technology, healthcare expectations, future usability of the program and overall satisfaction. Our results concur with other studies related to these focus areas (Marquis, 2015; Paneroni, 2015). One novel aspect of the survey our study conducted was addressing the relationship between participants where participants agreed that interacting with other participants benefitted their performance in the program. Some participants in our study needed additional assistance using the computer and interestingly other studies have identified the technology as being problematic at times (Hoaas et al., 2016; Paneroni, 2015). Another positive aspect was that participants stated that they would like to participate in telerehabilitation if the service continued which has also been shown previously (Paneroni et al., 2015).

The strength of this study is that we incorporated both quantitative and qualitative data with the latter methodology allowing for a greater depth of detail to emerge about the experiences that participants had with telerehabilitation. Only one previous study has incorporated both quantitative and qualitative methodology to evaluate perceptions of telerehabilitation in COPD (Hoaas et al., 2016). However, in this study the treating physiotherapist was present during focus groups while in the current study the assessor was independent of the study intervention, allowing participants to more freely give their opinions about the service. In the current study, four key themes were derived from the interviews with responses concurring with the quantitative data. The first theme was virtual interaction with technology. Firstly, the virtual interaction between physiotherapist and patients was discussed. Although this interaction was reported as satisfactory in the participant satisfaction survey, telerehabilitation compared to centre-based programs may be limited in not providing direct face-to-face interactions or any physical assistance from a physiotherapist. In the semi-structured interviews, participants reported that interacting through the computer screen was just like having the physiotherapist training them at home. Other studies using online group exercise sessions led by a physiotherapist also reported this interaction to be like exercising at a centre (Burkow, 2013; Taylor, 2011). Patient to patient virtual group interaction was another topic discussed in the interviews with participants acknowledging that they enjoyed exercising in a group and found it easier to exercise with others. This study concurs with other studies where participants reported the positive social aspect of exercising together and appreciating the shared experience (Burkow, 2013; Burkow, 2015; Taylor, 2011). It certainly seems that a combination of virtual group interaction with virtual physiotherapist supervision provides encouragement, support and motivation during exercise sessions.

The second and third themes which emerged from the interviews related to the use of equipment and the impact of technology on convenience. Participants who were still engaged in employment found the program particularly flexible and beneficial with the equipment being available at home to use at a time that suited their schedule. This finding is consistent with another study where participants were happy with the availability of training facilities at home (Hoaas et al., 2016). Our study also concurs with another study (Burkow et al., 2013) where participants have reported that it was a challenge to prepare and travel to the rehabilitation centre, and that participating at home is convenient and helps to conserve energy to exercise. In previous studies, problems with technology have been identified such as low quality of video or audio output, technology not being user friendly (Paneroni et al., 2015), and poor stability of the videoconferencing connection (Hoaas et al., 2016). In the current study, internet connection problems occurred for only a small number of participants. This problem was despite the metropolitan setting of the study allowing wireless 4G cellular data network from a telecommunication company. However, participants indicated the convenience of utilising this technology outweighed the disadvantage of internet connection problems since the service saved on costs and travel time. While these points of convenience may be even more critical to people living in rural and remote areas, internet coverage and connection are factors which future studies will need to address for a successful outcome.

The last theme which emerged from the interviews related to the perception of health benefits which the majority of participants described as positive. This positive perception of health benefits concurred with the major findings of the TeleR outcomes paper where an improvement in endurance exercise capacity and self-efficacy was shown for this group (Tsai et al., 2016). In another study, participants also reported that a long-term telerehabilitation program was effective and felt they were in good health with increased competence on performing daily tasks (Hoaas et al., 2016). One common factor between this study (Hoaas et al., 2016) and the current study was participants being highly motivated to exercise, which along with the increased fitness and perceived health benefits, probably contributed to the high levels of satisfaction reported across both studies.

A novel aspect of this study was the investigation of the participants’ partner’s satisfaction and experiences with telerehabilitation, which no other studies have previously reported. This group was also highly satisfied with the service although some partners were “neutral” in their responses about the reliability and ease of use of equipment. One other study has indicated the importance of involving “loved ones” in the management of people with COPD (Mequita et al., 2016) so it is important to identify the opinions of this group in order to ensure the effectiveness of home-based programs.

A limitation of this study was that not all telerehabilitation participants were interviewed for their opinions. However, participants were randomly chosen for this component of the data collection and the same themes emerged from the interviews that were found in the survey. A second limitation was that the semi-structured interviews were conducted following the completion of surveys. This order of events may have biased the participants to think within the framework of the themes highlighted in the survey as the themes emerging in the interviews were similar to that discussed in the surveys. However, one advantage of the use of the interviews was to allow participants to explore in more depth issues related to their satisfaction with the telerehabilitation program. A final limitation was that this study only reported on opinions of a short-term, eight-week supervised telerehabilitation exercise program so results are not translatable to long-term programs. This telerehabilitation program only incorporated an exercise component with no formal education so the patient experience of receiving education via videoconferencing technology cannot be determined.

## CONCLUSION

An eight-week supervised home-based telerehabilitation exercise program delivered in real-time via videoconferencing technology was well accepted by people with COPD with high satisfaction and positive interaction experiences as well as positive health benefits and convenience reported. Some participants identified difficulties with the internet connection but this did not impact on their overall satisfaction with the service.

## Figures and Tables

**Table 1:** Participants’ Characteristics

	TR group n=19	TR interviewed group n=11
**Age, years**	73 (8)	72 (8)
**Gender, male:female**	12:7	7:4
**Current smokers, n**	1	1
**BMI, kg/m2**	28 (4)	29 (4)
**Pulmonary function**		
FEV1 (L)	1.9 (2.2)	1.4 (0.5)
FEV1 (% predicted)	60 (23)	57 ( 20)
FVC (L)	2.6 (0.7)	2.5 (0.9)
FVC (% predicted)	89 (25)	79 (23)
FEV1/FVC (%)	51 (14)	54 (13)
TLC, % predicted	103 (30)	98 (34)
FRC, % predicted	115 (28)	113 (24)
RV, % predicted	133 (40)	128 (31)
RV/RLC ratio	52 (6)	52 (5)
DLCO, % predicted	56 (18)	60 (18)
**GOLD stage, n**		
I	5	2
II	6	3
III	8	5
IV	0	0
**Employment Status, %**		
Employed	26	27
Retired	74	73

Data are presented as mean (SD) unless otherwise stated.

TR, telerehabilitation; TR interviewed, participants in the telerehabilitation who completed semi-structured interviews; n, number; BMI, body mass index; kg, kilograms; m, metres; FEV_1_, forced expiratory volume in one second; L, litres; %, percentage. FVC, forced vital capacity; TLC, total lung capacity; FRC, functional residual capacity; RV, residual volume, RLC, residual lung capacity; DLCO, diffusing capacity for carbon monoxide; GOLD, Global Initiative for Chronic Obstructive Pulmonary Disease

**Table 2:** Results of Participant Satisfaction Survey (n=19)

	Yes, always (%)	Yes, often (%)	Yes, sometimes (%)	No, never (%)
**(i) Interactions via telerehabilitation**				

1. I could hear my physiotherapist during the telerehabilitation sessions	95	5	0	0

2. I could see my physiotherapist during the telerehabilitation sessions	95	5	0	0

3. I could speak to my physiotherapist during the telerehabilitation sessions	95	5	0	0

4. Interacting with other participants during the sessions benefitted my participation/performance in the program	74	0	21	5

	Strongly Agree; Agree (%)	Neutral (%)	Disagree; Strongly Disagree (%)	

**(ii) Equipment (Computer, bike and pulse oximeter)**				

5. I was able to use the computer and the computer program (VSee program)	100	0	0	
6. I was able to connect to the internet	95	0	5	
7. The computer equipment worked every time I used it	100	0	0	
8. I was able to use the pulse oximeter to check my oxygen saturation and heart rate	100	0	0	
9. I did not need any help while using the equipment (exercise or computer)	79	11	10	
10. Telerehabilitation exercise in my home was comfortable	100	0	0	
11. There was adequate space in my home for the exercise equipment (cycle, walking track)	95	5	0	
12. My privacy was respected during the telerehabilitation sessions	95	5	0	
13. The telerehabilitation education booklet provided useful information for me	100	0	0	

**(iii) Timeliness and convenience**				

14. Telerehabilitation saves me travel time, transport and parking costs	100	0	0	
15. Telerehabilitation scheduled appointments run on time	100	0	0	
16. Telerehabilitation provides me with convenient physiotherapy services	100	0	0	

**(iv) Level of satisfaction**				

17. I am satisfied with the quality of exercise sessions delivered using telerehabilitation	100	0	0	
18. I am satisfied with the information my physiotherapist provided about my COPD	100	0	0	
19. I would continue to participate in telerehabiliation if the service is available	79	21	0	
20. I would recommend the telerehabilitation service to my family or a friend	89	11	0	

n, number; %, percentage

**Table 3:** Results of Participants’ Partners Satisfaction Survey (n=7)

	Strongly Agree; Agree (%)	Neutral (%)	Disagree; Strongly Disagree (%)
1. Telerehabilitation saves the participant travel time.	100	0	0
2. Telerehabilitation saves the participant money on transport and parking costs.	100	0	0
3. Telerehabilitation provides the participant with the same exercise benefits at home as those received in a formal exercise centre.	100	0	0
4. The participant stayed motivated to exercise at home using telerehabilitation. services	100	0	0
5. The exercise equipment and computer provided were reliable and easy for the participant to use.	72	28	0
6. Telerehabilitation has made a positive difference to the participant’s lifestyle.	100	0	0
7. I would be comfortable for the participant to continue using telerehabilitation in the home.	100	0	0

n, number; %, percentage

**Table 4:** Quotes from the Semi-structured Interviews

Theme (a) Interactions through technology	
1	“She’s [physiotherapist] a professional. Got a professional in your home …… and you’re not talking to the computer, you’re talking to a person….it was really good, I enjoyed it.” (Male, 70 yrs)
2	“I think it’s almost the same you know, because you concentrate on your exercise not on the person you’re talking to. She gave very good instructions so there was no problem to do it.” (Female, 67 yrs)
3	“If we kind of met each other at least you were working on the same thing, we could encourage each other, I think that’s a good idea if we can do that, I think that’s great.” (Male, 82 yrs)
4	“There were three others and that didn’t worry me. It didn’t worry me at all. I was quite happy to do it that way……….. It was like a competition.” (Male, 70 yrs)
5	“I mean if she [the physiotherapist] had six other people I would’ve been fiddling about, waiting. So more than happy that I got personal treatment – though I couldn’t expect that all the time.” (Male, 70 yrs)

**Theme (b) Use of equipment**	

6	“Yeah it was dead easy, and the plug in that was easy, and the instructions were clear. And [the physiotherapist] left that [instruction book] with us, everybody had one of those. So … say you had a memory lapse or something, you just had to get the little booklet. It was all very, very clear.” (Female, 63 yrs)
7	“The whole set up regarding the computer and the bike and the system, was really really terrific. She set it up for me, she came. And they brought the bike here, set it up. And all I had to do was turn it on and turn it off.” (Male, 82 yrs)
8	“A couple of times it [the computer] froze…..and I hadn’t realised, I was walking and the phone went, and I thought I’m not going to answer that because otherwise it disrupts my walking. But of course it was [the physiotherapist]. She was telling me that….the screen was frozen and she ….. could no longer see me, and then of course when I looked at the screen I could see it was frozen.” (Female, 63 yrs)

**Theme (c) Convenience**	

9	“It fitted into my program perfectly…… … because it was in and out before nine o’clock and it’s good, I’d be here [at work] on duty at nine.” (Male, 70 yrs)
10	“The travel [to the hospital], I mean that’s two dollars fifty each time, that’s quite a big save you know? That’s quite a big save especially if I had to go three times a week” (Male, 82 yrs)
11	“From my point, I have to get dressed, sometimes I have trouble getting dressed, sometimes I have trouble having a shower because I lose my breath. So with that in mind, …..I’ve got to walk to the front door, walk down the lane and get the bus, and I’d probably have to stop two or three times. When it’s here [at home] I don’t have to do that. I’m not going to get puffed talking to [the physiotherapist], I’m only going to get puffed when she says get on the bike for fifteen minutes.” (Male, 70 yrs)
12	“[To attend the centre-based program] I’m away from about half past two to half past five, you know I don’t like leaving me wife that long.” (Male, 85 yrs)
13	“You don’t feel like exercising by the time you get to hospital because the trip has made you so breathless already that you’re kind of worn out.” (Male, 70 yrs).

**Theme (d) Health benefits**	

14	“The thing is when you do things for yourself you tend to get tired, take things a bit easy or you know, I’m done my early exercise blah blah, I’ll only walked five minutes today – but when you’ve got her [physiotherapist] on you, there’s no shortcuts”. (Male, 82 yrs)
15	“[What did you find most beneficial?] Well the health benefits I guess. I enjoyed seeing [the physiotherapist] three mornings a week. [The] motivation of having someone by appointment be around even though they’re not here, but they’re as good as here”. (Male, 70 yrs)
16	“Well I think you’ve got the impression that I’m a totally satisfied customer! I feel I benefited from the trial, I’m pleased that [the] hospital have made the facility available to me. Very grateful”. (Male, 70 yrs)
17	“I thought it was all good because it was specifically designed for our kind of problem, so I thought it was good. Sorry I’d like to find something a little bit negative, but I can’t. It was really good”. (Female, 63 yrs)

yrs, years old

## APPENDIX B TELEREHABILITATION SEMI-STRUCTURED INTERVIEW

1. Which part of the Telerehabilitation intervention worked well for you? ***Probes:***
  - How did you find using the computer?
  - How did you find using the exercise bike at home? Did you have adequate space at home?
  - How did you find using the internet USB?
  - How did you find using the finger pulse oximeter?
2. What was the best part of Telerehabilitation? ***Probes:***
  - On equipment: Computer, exercise bike, internet USB, pulse oximeter?
  - On convenience: interactions with the physiotherapist, time and travel cost (leading to next two questions)
3. What did you think about the interaction with the physiotherapist via the computer? ***Probes:***
  - Can you hear/speak/see your therapist easily?
  - Can you communicate with your therapist at ease? If it’s hard to communicate, why?
4. Did Telerehabilitation impact on your cost of living? ***Probes:***
  - Parking cost, Petrol cost, Travel time, scheduled appointment with your physiotherapist
5. Which part of the Telerehabilitation intervention didn’t work for you? ***Probes:***
  - How did you find using the computer?
  - How did you find using the exercise bike at home?
  - Internet connection problems?
  - Set up and space at home?
6. What did you like the least about Telerehabilitation?
  - Computer
  - Bike
  - Internet connection
  - Pulse oximeter
7. Overall, how satisfied are you with Telerehabilitation program?
8. Do you think the Telerehabilitation program would work in rural or remote areas? ***Probes:***
  - What adjustments need to be made to the Telerehabilitation program to make it more appropriate for the needs of your peers?
  - people with low health literacy, lower social economics status, culturally and linguistically diverse communities, Aboriginal and Torres Strait Island people, other harder to reach groups
9. Is there anything else that you would like to add?
